# Prevalence of Bladder Cancers Incidentally Detected During Multiparametric MRI Scans of the Prostate Gland and the Clinical Significance of Scoring Them According to VI-RADS: A Pictorial Single-Centre Study

**DOI:** 10.5334/jbsr.3318

**Published:** 2024-02-01

**Authors:** Ramazan Orkun Onder, Serdar Aslan, Tümay Bekci

**Affiliations:** 1 Faculty of Medicine, Department of Radiology, Giresun University, Giresun, Turkey; 2 Faculty of Medicine, Department of Radiology, Giresun University, Giresun, Turkey; 3 Faculty of Medicine, Department of Radiology, Giresun University, Giresun, Turkey

**Keywords:** Multiparametric prostate MRI, incidental, bladder cancer, VI-RADS

## Abstract

**Purpose:**

To determine the prevalence of incidentally detected bladder cancers (BCs) on multiparametric magnetic resonance imaging (mpMRI) of the prostate and to highlight the clinical importance of scoring them according to the Vesical Imaging-Reporting and Data System (VI-RADS).

**Materials and Methods:**

VI-RADS scores for incidental bladder lesions on mpMRI of the prostate were collected in 1693 patients with elevated prostate-specific antigen but no hematuria. The study included 19 patients with 28 incidental bladder lesions.

**Results:**

During this period, 39 incidental bladder lesions were found in 30 patients, representing 1.7% of cases. Of the 28 lesions, 11 were categorized by VI-RADS as VI-RADS 1, 14 as VI-RADS 2, 1 as VI-RADS 3, 1 as VI-RADS 4, and 1 as VI-RADS 5. Histopathological examination revealed 1 benign lesion, 24 non-muscle invasive BCs, and 3 muscle-invasive BCs in the 19 patients. Impressively, 97% of the incidental lesions detected by prostate mpMRI and categorized by VI-RADS were BCs without apparent prostate cancer invasion. Notably, 93% of these lesions were consistent with histopathological findings of muscle invasion and extravesical spread.

**Conclusion:**

Our study concludes the prevalence 1% incidental BC in prostate mpMRI. The research underscores a thorough bladder examination during prostate MRI scans. Utilizing mpMRI assists in distinguishing varying BC stages, aiding treatment decisions, and patient outcomes. VI-RADS categorization aligns with histopathological results, enhancing diagnosis, and healthcare communication. Early detection significantly influences patient care by enabling timely interventions and suitable treatment strategies, particularly for low-stage BCs linked to reduced progression and recurrence rates.

## Introduction

Prostate cancer has the highest prevalence among malignancies in men and is the second leading cause of cancer-related death [1]. After the widespread introduction of prostate-specific antigen (PSA) testing, there was an initial increase in prostate cancer rates, which then decreased and stabilized [2]. The widespread use of multiparametric magnetic resonance imaging (mpMRI) for prostate examination has improved the detection, localization, and staging of prostate cancer [3].

Bladder cancer (BC) is an important public health problem with high morbidity and mortality rates [4]. It is estimated that there will be 82,290 new cases of BC among American patients in 2023 and an estimated 16,710 deaths due to this disease [5]. However, there are no recognized screening tests for BC. Diagnosis is usually made after symptomatic presentation following hematuria, the most common symptom that triggers evaluation [6]. While the primary diagnostic focus is usually prostate cancer in men, with the increasing prevalence of prostate mpMRI, the likelihood of incidental detection of other pelvic pathologies, including BC, has increased. These incidental findings provide an opportunity for early diagnosis and potentially better patient outcomes [7].

mpMRI is highly effective in the local and nodal staging of BC due to its exceptional soft tissue contrast. It highlights T2-weighted imaging (WI) and enables differentiation of bladder wall layers, distinguishing non-muscle-invasive bladder cancer (NMIBC) from muscle-invasive bladder cancer (MIBC) [8]. Clinical management involves distinguishing NMIBC and MIBC. NMIBC is treated with transurethral resection or intravesical therapy like mitomycin Cor Bacillus Calmette–Guerin (BCG). MIBC treatment includes cystectomy, radiation, or chemotherapy [9].

The Vesical Imaging-Reporting and Data System (VI-RADS) is a standardized reporting system for the interpretation and reporting of BC imaging. The aim of VI-RADS is to provide a structured and consistent approach for interpreting BC imaging studies by facilitating communication among radiologists, urologists, and oncologists [10]. A five-point VI-RADS score was created using the categories of T2-WI, dynamic contrast enhancement (DCE), and diffusion-weighted MRI, which indicates the likelihood of muscle invasion. VI-RADS scores are shown in Table 1. Thus, it aims to increase the accuracy and reliability of BC diagnosis, staging, and treatment planning [11].

**Table 1:** VI-RADS scores

VI-RADS 1	It is highly improbable that there is any muscular invasion
VI-RADS 2	The presence of muscle invasion is doubtful
VI-RADS 3	The existence of muscle invasion is ambiguous
VI-RADS 4	There is a high probability of muscle invasion
VI-RADS 5	Muscle invasion and beyond the bladder is highly likely

The prevalence of incidentally detected BCs on mpMRI of the prostate has been studied to a limited extent, and the scoring of these findings according to VI-RADS has not been adequately investigated. The aim of this study was to determine the prevalence of incidental BCs on mpMRI of the prostate and to improve the understanding and management of incidental BCs by highlighting the clinical importance of VI-RADS scoring.

## Materials and Methods

### Ethics

This study was approved by the Institutional Ethics Committee. The requirement for informed consent was waived. The study protocol conforms to the ethical guidelines of the 1975 Declaration of Helsinki.

### Study population

Between December 2019 and July 2023, VI-RADS scores of bladder lesions detected incidentally on mpMRI scans of the prostate were recorded in 1693 patients with high PSA levels but no hematuria. The study focused on demographics, imaging details, and histopathological information such as age, lesion count, largest diameter of lesion on mpMRI, VI-RADS score, tumor characteristics, grade, and treatment (Tables 2 and 3). Only patients treated at the center were included, excluding those with bladder-invasive prostate cancer. In our study, we retrospectively analyzed prospectively collected data from 19 patients with 28 incidental bladder lesions. Cases where the origin of such lesions couldn’t be definitively linked to prostate cancer metastasis underwent cystoscopy followed by biopsy and/or TURBT, as determined during the cystoscopic examination.

**Table 2:** Demographic data

Age (years), mean	67, 8
Smoking history, *n* (%)	
Yes No	10 (52)
	9 (48)
Number of *n*	
Patients Lesions	19
	28

**Table 3:** Clinical characteristics of patients with incidental bladder lesions

LARGEST LESION DIAMETER ONMPMRI (CM)	HISTOPATHOLOGY			
	VI-RADS SCORE	STAGE	GRADE	TREATMENT
1.1	2	Ta	Low	TURBT
1.5	2	Ta	High	TURBT+BCG
0.7	1	Ta	Low	TURBT
0.8	1	Ta	Low	TURBT
0.7	1	Ta	Low	TURBT
0.9	1	Ta	Low	TURBT
1.2	2	Ta	Low	TURBT
1.1	2	Ta	Low	TURBT
1.4	2	Ta	Low	TURBT
1.7	2	Ta	Low	TURBT
1.5	2	Ta	Low	TURBT
1.9	2	Ta	Low	TURBT
0.7	1	Ta	Low	TURBT
2.1	3	T2, (SCNC)	High	Radical Cystectomy
1.1	4	T2	High	Radical Cystectomy
1.3	2	Ta	Low	TURBT
1.2	2	Ta	Low	TURBT
2	2	Ta	Low	TURBT
1.5	2	Ta	Low	TURBT
3.5	2	T1	High	TURBT + BCG
0.6	1	Ta	Low	TURBT
0.9	1	Ta	Low	TURBT
0.8	1	Ta	Low	TURBT
0.7	1	Ta	Low	TURBT
0.8	1	Ta	Low	TURBT
2.8	5	T2	High	Radical Cystectomy
0.5	1	Benign (Inflamed urothelial tissue)		-
1.5	2	Ta	Low	TURBT

SCNC, small cell neuroendocrine carcinoma; TURBT, transurethral resection of bladder tumor; BCG, Bacillus Calmette–Guerin.

### Image acquisition

All MRI examinations were performed using a 1.5-T MRI system (Magnetom Symphony; Siemens Medical Solutions, Erlangen, Germany) in the supine position using a surface-phased array endorectal coil. Axial, sagittal, and coronal T2-weighted sequences oriented with respect to the main axis of the prostate were acquired. Diffusion-weighted imaging (DWI) was performed using single-shot echoplanar sequences (EPI) with a maximum *b* value of 1,400 s/mm^2^. In addition, T1-weighted axial sequence imaging was performed by intravenous administration of 1 mmol/kg gadobutrol (Gadovist®, Bayer Healthcare, Germany), a gadolinium-based contrast agent, at a rate of 3 mL/sec followed by an infusion of 15 mL of saline solution.

## Results

**Figure 1:**
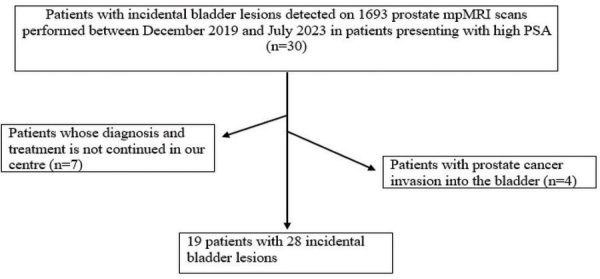
Flowchart of patient selection.

**Figure 2:**
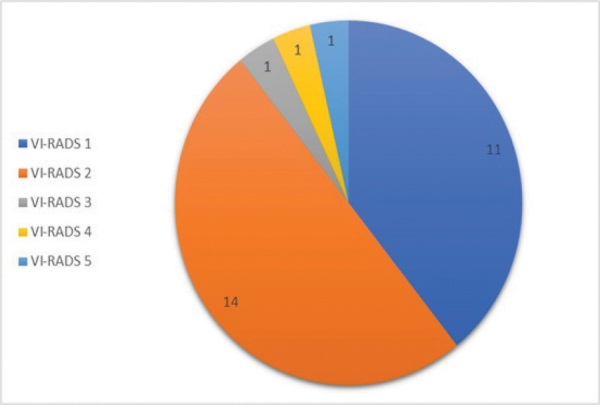
VI-RADS score distribution of incidental bladder lesions.

**Figure 3:**
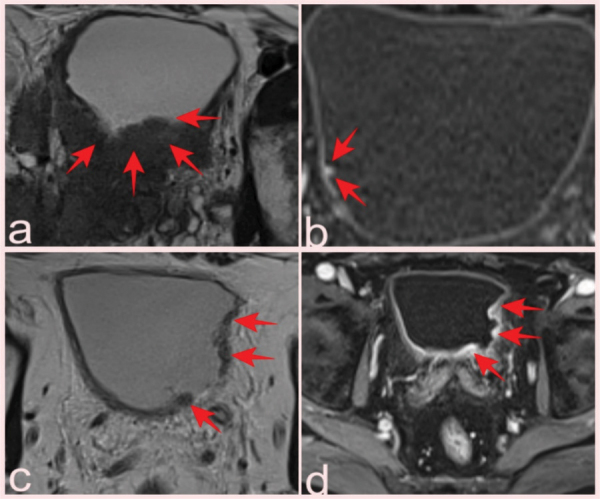
(a) Hypointense soft tissue showing bladder invasion on axial T2-WI in a 74-year-old man with Gleason score 3+4 prostate cancer (arrows). (b) Prostate mpMRI in a 66-year-old patient showed a 0.5 cm lesion on the right posterolateral wall of the bladder that was missed on conventional images but was detected by contrast uptake on DCE images and reported as VI-RADS 1; pathology was benign (Inflamed urothelial tissue) (arrows). T2-WI (c) and DCE (d) images of a 64-year-old patient with a high-grade pT2 tumor classified as VI-RADS 4 (arrows).

**Figure 4:**
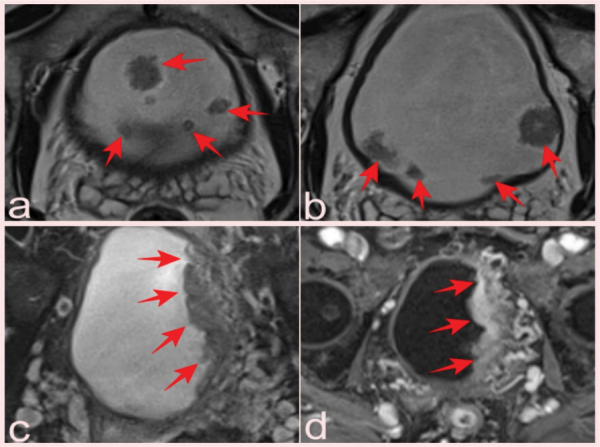
(a, b) Two slices from T2-WI of a 69-year-old patient who presented with elevated PSA and had a total of 10 lesions with pTa pathology, four of which were reported as VI-RADS 1 and six as VIRADS 2 (arrows). Fat-suppressed T2-WI (c) and (d) DCE images of a 75-year-old man with a serum PSA value of 8.9 ng/mL and Gleason score of 3 + 3 prostate cancer, with a lesion reported as VI-RADS 5 but pathologically as a high-grade pT2 tumor (arrows).

**Figure 5:**
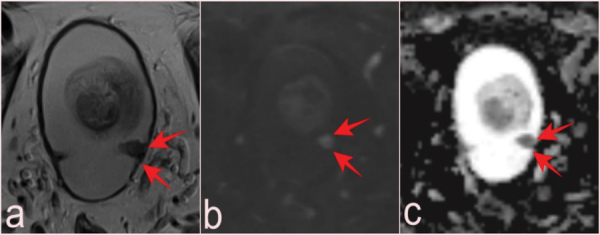
T2-WI (a), DWI (b), and ADC map (c) of a lesion with low-grade pTa tumor pathology reported as VI-RADS 1, which can be missed at the level of mucosal folds in a 69-year-old patient with prostate mpMRI after PSA elevation (arrows). ADC, apparent diffusion coefficient.

**Figure 6:**
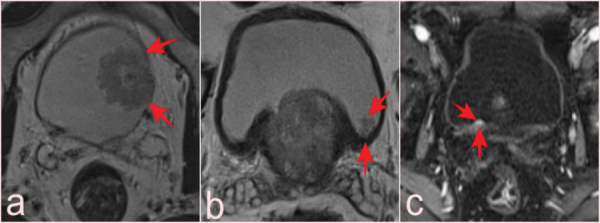
(a) T2-WI shows a VI-RADS 2 lesion with a high-grade pT1 tumor in a 74-year-old patient (arrows). (b) In a 60-year-old patient, a T2-WI image revealed a VI-RADS 1 lesion, 0.8 cm, missed without careful left posterior wall examination; pathology was pTa tumor (arrows). (c) VI-RADS 1 lesion, 0.6 cm, on the right posterior wall of a 74-year-old patient; contrast-enhanced DCE images aid selection; pathology: pTa tumor (arrows).

**Figure 7:**
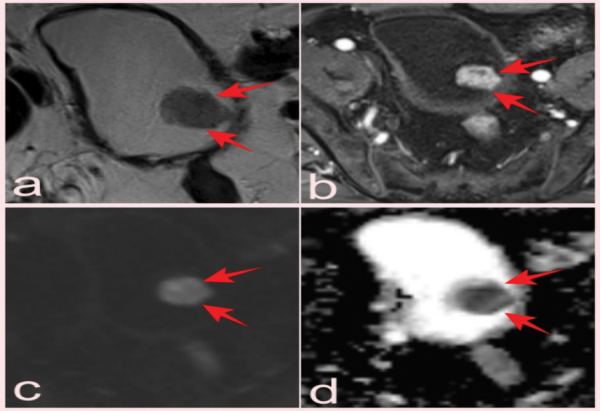
T2-WI (a), DCE images (b), DWI (c), and (d) ADC map of an incidental bladder lesion reported as VI-RADS 3 but pathologically diagnosed as high-grade pT2 small-cell neuroendocrine carcinoma on prostate mpMRI performed after elevated PSA in a 69-year-old patient (arrows).

**Figure 8:**
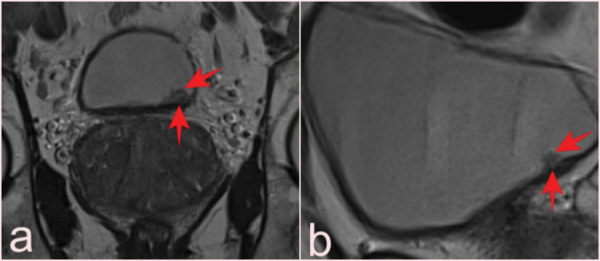
T2-WI of incidental bladder lesions detected only on coronal slices (a) in a 65-year-old patient, reported as VI-RADS 1, 0.7 cm in size and with pTa tumor pathology, and in sagittal slices (b) only in a 55-year-old patient, reported as VI-RADS 1, 0.8 cm in size and with pTa tumor pathology (arrows).

A total of 39 incidental bladder lesions were detected in 30 patients (1.7%) out of 1693 prostate mpMRI scans. Seven of the 30 patients did not continue their diagnosis and treatment at our center. Prostate cancer invasion of the bladder was suspected in 4 of 23 patients with incidental bladder lesions, and biopsy confirmed prostate adenocarcinoma invasion. In 19 patients without suspected bladder-invasive prostate cancer, 28 incidental bladder lesions were detected, including 10 lesions in 1 patient. As a result, the study group consisted of 19 patients with a total of 28 bladder lesions. Figure 1 shows the patient selection. The mean age of these patients was 67.8 years, the oldest was 86 years, and the youngest was 55 years. 68% of the patients were over 65 years of age, and 52% were smokers. Bladder lesions ranged in diameter from 0.5 cm to 3.5 cm. Of the 28 bladder lesions, 11 were reported as VI-RADS 1 (40%), 14 as VI-RADS 2 (50%), 1 as VI-RADS 3 (3.3%), 1 as VI-RADS 4 (3.3%), and 1 as VI-RADS 5 (3.3%) (Figure 2). The 28 bladder lesions were visualized using cystoscopy. Sixteen of the 19 patients (85%) underwent TURBT, and 3 (15%) underwent radical cystectomy. One bladder lesion reported as VI-RADS 1 showed benign features (inflamed urothelial tissue) on pathological examination. In the pathology of the other 24 bladder lesions reported as VI-RADS 1 and VI-RADS 2, 23 had pTa urothelial carcinoma (22 low-grade and 1 high-grade) and 1 had high-grade pT1 urothelial carcinoma. The pathology of the bladder lesion reported as VI-RADS 3 was high-grade pT2 invasive small-cell neuroendocrine carcinoma. The pathology of the bladder lesion, reported as VI-RADS 4, was pT2 invasive urothelial carcinoma. The pathology of the bladder lesion reported as VI-RADS 5, which was thought to have muscle invasion and extravesical extension, was a pT2 invasive urothelial carcinoma. As a result, histopathological data showed that 1 (4%) of the 28 bladder lesions was benign, 24 (85%) were pta-pT1 (NMIBC), and 3 (11%) were pT2 (MIBC). In addition, 5 (18%) BC were high-grade and 22 (79%) were low-grade. In other words, BC was detected in 18 of 19 patients (95%). Fifteen (85%) patients had pTa-PT1 (NMIBC) and 3 (15%) patients had pT2(MIBC). In conclusion, our study showed that the prevalence of incidental BC was 1% (18 patients) in 1693 patients who underwent prostate mpMRI. Of the incidental bladder lesions reported on prostate mpMRI without suspicion of prostate cancer invasion into the bladder, and categorized by VI-RADS, 97% were BC. Furthermore, 26 out of these 28 bladder lesions (93%) were found to be concordant with the histopathological data, indicating muscle invasion and extravesical spread. The mpMRI findings of some of the patients are shown in Figures 3 to 8. All patients were treated with standard TURBT, TURBT with intravesical BCG, or radical cystectomy. None of the patients with low-grade and NMIBC relapsed during a median follow-up of 18 months.

## Discussion

BC, for which there is no specific screening test, is the second most common genitourinary malignancy after prostate cancer, with more than half a million new cases and 200,000 deaths reported worldwide annually [12]. The increasing use of mpMRI in prostate screening in recent years has led to an increased likelihood of incidental bladder lesion detection, providing an opportunity for early diagnosis and potentially better patient outcomes [13]. In our study, incidental bladder lesions (30 patients, 1.7%) and incidental BC (18 patients, 1%) were found in a significant proportion of the 1,693 patients who underwent prostate mpMRI. In a retrospective study conducted by Rayn et al. in 2018, these rates were 0.8% and 4%, respectively [14]. In addition, in another retrospective study conducted by Wagnerova et al. in 2023, 2.4% of extraprostatic findings were found to be clinically significant [15]. In our study, the majority of incidentally detected bladder lesions were low-grade (79%) and non-muscle-invasive lesions (85%). Our study showed that the majority (93%) of incidental bladder lesions categorized by the VI-RADS were consistent with histopathological data indicating muscle invasion. This reinforces the utility of VI-RADS in improving the accuracy and reliability of BC diagnosis, staging, and treatment planning, which may lead to more effective patient management as a result of early detection and characterization of incidental BCs [16]. The clinical utility of mpMRI in the local and nodal staging of BC was clearly demonstrated in our study. The ability of mpMRI to accurately discriminate between NMIBC and MIBC is crucial, as it guides treatment decisions [17].

Another important point is that the bladder is usually not fully visualized on axial slices in mpMRI of the prostate. In our study, there were lesions that were not visualized on axial slices but were detected on coronal and sagittal slices (Figure 8). Therefore, all planes and sequences should be examined in mpMRI.

Although our study provides valuable information on the prevalence and characterization of incidental BC detected by mpMRI of the prostate, it is not without limitations. The relatively small sample size, the fact that the study was single-center and only applicable to men, and the fact that not all patients were treated at our center may limit the generalizability of our findings.

In conclusion, identifying BC incidentally on a prostate mpMRI can have significant clinical implications. This is particularly true for cases presenting with low-stage and non-muscle-invasive tumors, as they tend to exhibit considerably lower rates of progression, recurrence, and mortality. Therefore, during prostate mpMRI scans, the prostate gland as well as the bladder should be examined in detail in all planes and sequences, and incidental bladder lesions should be scored and reported according to VI-RADS.
